# Targeting Interleukin-2-Inducible T-Cell Kinase (ITK) Differentiates GVL and GVHD in Allo-HSCT

**DOI:** 10.3389/fimmu.2020.593863

**Published:** 2020-11-26

**Authors:** Mahinbanu Mammadli, Weishan Huang, Rebecca Harris, Aisha Sultana, Ying Cheng, Wei Tong, Jeffery Pu, Teresa Gentile, Shanti Dsouza, Qi Yang, Alaji Bah, Avery August, Mobin Karimi

**Affiliations:** ^1^ Department of Microbiology and Immunology, SUNY Upstate Medical University, Syracuse, NY, United States; ^2^ Department of Pathobiological Sciences, School of Veterinary Medicine, Louisiana State University, Baton Rouge, LA, United States; ^3^ Department of Microbiology and Immunology, College of Veterinary Medicine, Cornell University, Ithaca, NY, United States; ^4^ Division of Hematology, Children’s Hospital of Philadelphia, Philadelphia, PA, United States; ^5^ Department of Hematology, SUNY Upstate Medical University, Syracuse, NY, United States; ^6^ Department of Immunology and Microbial Disease, Albany Medical College, Albany, NY, United States; ^7^ Department of Biochemistry and Molecular Biology, SUNY Upstate Medical University, Syracuse, NY, United States

**Keywords:** GVHD after blood transfusion, T cell, GvL, ITK deficiency, Eomesodermin (EOMES), JAK-STAT signalling pathway

## Abstract

Allogeneic hematopoietic stem cell transplantation is a potentially curative procedure for many malignant diseases. Donor T cells prevent disease recurrence *via* graft-versus-leukemia (GVL) effect. Donor T cells also contribute to graft-versus-host disease (GVHD), a debilitating and potentially fatal complication. Novel treatment strategies are needed which allow preservation of GVL effects without causing GVHD. Using murine models, we show that targeting IL-2-inducible T cell kinase (ITK) in donor T cells reduces GVHD while preserving GVL effects. Both CD8^+^ and CD4^+^ donor T cells from *Itk^-/-^* mice produce less inflammatory cytokines and show decrease migration to GVHD target organs such as the liver and small intestine, while maintaining GVL efficacy against primary B-cell acute lymphoblastic leukemia (B-ALL). *Itk^-/-^* T cells exhibit reduced expression of IRF4 and decreased JAK/STAT signaling activity but upregulating expression of Eomesodermin (Eomes) and preserve cytotoxicity, necessary for GVL effect. Transcriptome analysis indicates that ITK signaling controls chemokine receptor expression during alloactivation, which in turn affects the ability of donor T cells to migrate to GVHD target organs. Our data suggest that inhibiting ITK could be a therapeutic strategy to reduce GVHD while preserving the beneficial GVL effects following allo-HSCT treatment.

## Highlights

- ITK-deficient donor T cells exhibit minimal GVHD, but maintain GVL activity.
- ITK-deficient donor T cells exhibit significantly reduced production of inflammatory cytokines and migration to GVHD target organs.
- Eomes is required for the GVL effect.

## Introduction

During allogeneic hematopoietic stem cell transplantation (allo-HSCT), alloreactive donor T cells are essential for the graft-versus leukemia effect (GVL) (1–3). The same donor T cells may also cause significant tissue damage to the host, known as graft-versus-host disease (GVHD) (4). Development of GVHD results in significant morbidity and mortality which complicates allo-HSCT, a potentially curative treatment for leukemia. Standard immunosuppressive therapy for GVHD is often therapeutically sub-optimal and predisposes patients to opportunistic infections such as Cytomegalovirus (CMV) and relapse of the underlying malignancy (5, 6). Thus, specific signaling pathways that can be targeted to allow the effects of GVL to persist while inhibiting GVHD need to be identified. The Tec family nonreceptor tyrosine kinase, Interleukin-2-inducible T cell kinase (ITK), regulates activation of T cells downstream of the T cell receptor (TCR). ITK is involved in the activation of intracellular calcium signaling and MAPK pathways, as well as polarization of the actin cytoskeleton, supporting an integral role for ITK in T cell activation and function (7, 8). ITK is involved in signaling which leads to cytokine production by T cell populations, and also negatively regulates the development of a distinct, innate-type cytokine-producing T cell population in the thymus (9), referred to as innate memory phenotype (IMP) T cells. These cells express significantly higher levels of CD122, CD44, and Eomes compared to T cells from WT mice. Since the activation, expansion, cytokine production, and migration of alloreactive donor T cells to target organs are hallmarks of GVHD (10, 11), and ITK is involved in these T cell activities, we examined the role of ITK in GVHD and GVL in an allo-HSCT model.

Previous studies have shown that Ibrutinib, an inhibitor of the related Tec kinase Bruton’s tyrosine kinase (BTK) which can also inhibit ITK, is able to reduce chronic GVHD (12). Here we use a murine model of allo-HSCT involving allotransplant of T cells from C57Bl/6 (WT) mice or *Itk^-/-^* mice into BALB/c mice, to examine GVHD and GVL. We found that CD4^+^ and CD8^+^ T cells transplanted from ITK-signaling-deficient mice induce significantly less GVHD while retaining GVL function, compared to T cells from WT mice. We also found that this separation of GVHD from GVL was not dependent on the development of IMP T cells since T cells from IL-4 receptor-alpha and ITK-double knockout mice (*Itk/Il4ra* DKO), which lack the IMP phenotype (13), did not induce GVHD, Instead, the presence or absence of ITK separated GVHD from GVL in a cell-intrinsic manner. Furthermore, *Itk^-/-^* donor T cells exhibited cell-intrinsic reduction in proliferation, and both CD8^+^ and CD4^+^ T cells donor T cells from *Itk^-/-^* mice exhibit increased expression of perforin and significantly reduced expression of pro-inflammatory cytokines. Both CD4^+^ and CD8^+^ T cells from *Itk^-/-^* mice upregulate the key transcription factor Eomes, which we found is critical for both GVHD and GVL, since *Itk^-/-^* Eomes^flox/flox^ CD4cre^+^ T cell donors (deficient in both Eomes and ITK) did not mount a cytotoxic response against primary leukemia cells or clear tumor cells, both *in vitro* and *in vivo*. Our data further demonstrate that ITK deficiency affects JAK1/2 (14) and IRF-4 (15) signaling, and CD4^+^ and CD8^+^ T cells from ITK-deficient mice show defects in T cell migration into GVHD target tissues, caused by reduced expression of chemokine receptors. This leads to decreased tissue damage during allo-HSCT. *Itk^-/-^* T cells can successfully clear leukemia cells in circulation, however they are unable to clear subcutaneously growing leukemic cells due to this migration defect. Finally, RNA sequencing data revealed that ITK deficiency impacts genes involved in cytokine production, cell adhesion, and chemokine and cytokine receptor expression. These genes are involved in the pathogenesis of GVHD. Our studies identify a specific and novel potential therapeutic target and its downstream mechanism for separating GVHD and GVL after allo-HSCT. Targeting ITK may also prove beneficial for other T cell-mediated diseases.

## Materials and Methods

### Mice

*Itk^-/-^* mice were described previously (16). C57BL/6, C57BL/6.SJL (B6-SJL), ROSA26-pCAGGs-LSL-Luciferase, Thy1.1 (B6.PL-Thy1a/CyJ), CD45.1 (B6.SJL-Ptprc^a^ Pepc^b^/BoyJ), and BALB/c mice were purchased from Charles River or Jackson Laboratory. Eomes^flox/flox^ mice, B6.129S1mice, and CD4cre mice were purchased from Jackson Laboratory. Mice expressing Cre driven by the CMV promoter (CMV-Cre) were purchased from the Jackson Laboratory and crossed to ROSA26-pCAGGs-LSL-Luciferase mice (B6-luc). B6-luc mice were bred with *Itk^-/-^* mice to create *Itk^-/-^* luc mice. *Itk^-/-^*/*Il4ra^-^* ^/-^ double knockout mice have been described (13). Mice aged 8–12 weeks were used, and all experiments were performed with age and sex-matched mice. Animal maintenance and experimentation were performed in accordance with the rules and guidance set by the institutional animal care and use committees at SUNY Upstate Medical University and Cornell University.

### Reagents, Cell Lines, Flow Cytometry

Monoclonal antibodies were purchased from eBiosciences (San Diego, CA) or BD Biosciences (San Diego, CA). Antibodies used included anti-CD3, anti-CD28, anti-CD3-FITC, Anti-CD3-BV605, anti-CD8-FITC, anti-BrdU-APC, anti-IFNγ-APC, anti-TNFα-PE, anti-CD45.1-PerCPCy5.5, anti-CD122-APC, anti-CD44-Pacific blue, anti-Eomes-PE-Cy7, anti-CD25-BV421, anti-FoxP3-APC, anti-T-bet-BV421, anti-CD4-BV785, anti-CD45.1-Pacific Blue, and anti-H-2K^d^-Pacific Blue. We performed multiplex ELISAs using Biolegend LEGENDplex kits, and some kits were custom ordered to detect both mouse and human cytokines. Luciferin was purchased from Perkin Elmer (Waltham, MA) and Gold Bio (St Louis MO). Dead cells were excluded from analysis with LIVE/DEAD Fixable Aqua Dead Cell staining. Flow cytometry was performed using a BD LSR-II or BD LSRFortessa cytometer (BD Biosciences). Data were analyzed with FlowJo software (Tree Star, Ashland, OR).

For cell sorting, T cells were purified with either anti-CD8 or anti-CD4 magnetic beads using MACS columns (Miltenyi Biotec, Auburn, CA) prior to cell surface staining. FACS sorting was performed with a FACS Aria III cell sorter (BD Biosciences). FACS-sorted populations were typically of >95% purity. Antibodies against IRF4, STAT3, JAK2, JAK1, GAPDH, and β-Actin (for total and/or phospho proteins) were purchased from Cell Signaling Technology (Danvers, MA). All cell culture reagents and chemicals were purchased from Invitrogen (Grand Island, NY) and Sigma-Aldrich (St. Louis, MO), unless otherwise specified. The A20 cell lines (American Type Culture Collection; Manassas, VA), and primary mouse B-ALL blast cells (17) were transduced with luciferase, and cultured as described previously (18).

### Allo-HSCT and GVL Studies

Lethally irradiated BALB/c mice (800 cGy) were injected intravenously with 10 × 10^6^ T cell-depleted bone marrow (_TCD_BM) cells with or without 1 × 10^6^ FACS-sorted CD8^+^ T cells, 1 × 10^6^ CD4^+^ T cells, or CD8/CD4 cells mixed at a 1:1 ratio. FACS-sorted total CD8^+^, total CD4^+^, or mixed donor CD4^+^ and CD8^+^ T cells from WT (C57Bl/6) or *Itk^-/-^* mice were used. For GVL experiments, B-cell acute lymphoblastic leukemia (B-ALL) primary blasts (17) transduced with luciferase were cultured as described previously (18), and 2 × 10^5^ luciferase-expressing B-ALL blasts were used. Mice were evaluated twice a week from the time of leukemia cell injection for 65 days by bioluminescence imaging using the IVIS 50 Imaging System (Xenogen) as previously described (19). Clinical presentation of the mice was assessed 2–3 times per week by a scoring system that sums changes in five clinical parameters: weight loss, posture, activity, fur texture, and skin integrity (20). Mice were euthanized when they lost ≥30% of their initial body weight or became moribund.

For chimera experiments, bone marrow cells from *Itk^-/-^* (CD45.1^+^) or C57Bl/6 (CD45.2^+^) mice were mixed at different ratios—1:1 (WT:*Itk^-/-^*), 1:2, 1:3, or 1:4—and transplanted into lethally irradiated Thy1.1 mice. In some experiments, we used *Itk^-/-^* on a CD45.2 background and WT on a CD45.1 background as indicated in the figure legends. Mice were bled from the tail vein after 9 weeks to determine the presence of *Itk* ^-/-^ and WT cells. For GVHD assessment experiments, *Itk* ^-/-^ (CD45.1^+^) and WT (CD45.2) T cells were FACS-sorted from Thy1.1 hosts and then transplanted to irradiated BALB/c mice carrying leukemia cells, along with _TCD_BM as described above. This was followed by analysis of GVHD and GVL. In some experiments FACS-sorted CD8^+^ T cells from WT or *Itk^-/-^* mice were mixed at a 1:1 ratio and injected into BALB/c mice (2 × 10^6^ CD8^+^ T cells total).

### Tissues Imaging

Allo-HSCT was performed with 10 × 10^6^ WT T cell-depleted BM cells and 1 × 10^6^ FACS-sorted CD8^+^ or 1 × 10^6^ FACS-sorted CD4^+^ T cells (from B6-luc or *Itk* ^-/-^ *luc* mice) and bioluminescence imaging of tissues was performed as previously described (21). Briefly, 5 min after injection with luciferin (10 μg/g body weight), selected tissues were prepared and imaged for 5 min. Imaging data were analyzed and quantified with Living Image Software (Xenogen) and Igor Pro (Wave Metrics, Lake Oswego, OR).

### Cytokine Production, Cytotoxicity, and BrdU Incorporation Assays

On Day 7 post-transplantation, serum and single cell suspensions of spleens were obtained. Serum IL-33, IL-1α, IFN-γ, TNF-α and IL-17A content was determined by multiplex cytokine assays (Biolegend LEGENDplex). T cells were stimulated with anti-CD3/anti-CD28 for 4–6 h in the presence of brefeldin A (10 μM) and stained intracellularly for cytokines (IFN-γ and TNF-α). Control cells were stimulated with PMA and ionomycin in the presence of brefeldin A.

### Proliferation Assays

For detection of BrdU, mice were given BrdU with an initial bolus of BrdU (2 mg per 200 μl intraperitoneally) and drinking water containing BrdU (1 mg/ml) for 2 days. BrDU incorporation was performed using BrDU kit (Invitrogen) according to the manufacturer’s instructions.

### Cytotoxicity Assays

For cytotoxicity assays, luciferase-expressing A20 cells were seeded in 96-well flat bottom plates at a concentration of 3x10^5^ cells/ml. D-firefly luciferin potassium salt (75 μg/ml; Caliper Hopkinton, MA) was added to each well and bioluminescence was measured with the IVIS 50 Imaging System. Subsequently, ex vivo effector cells (MACS-sorted or FACS-sorted CD8^+^ T cells from bone marrow-transplanted mice) were added at 40:1, 10:1, and 5:1 effector-to-target (E:T) ratios and incubated at 37°C for 4 h. Bioluminescence in relative luciferase units (RLU) was then measured for 1 min. Cells treated with 1% Nonidet P-40 was used as a measure of maximal killing. Target cells incubated without effector cells were used to measure spontaneous death. Triplicate wells were averaged and percent lysis was calculated from the data using the following equation: % specific lysis = 100 × (spontaneous death RLU–test RLU)/(spontaneous death RLU – maximal killing RLU) (22).

### Migration Assays

Lethally irradiated BALB/c mice were injected intravenously with 10 × 10^6^ WT _TCD_BM cells from B6.PL-*Thy1^a^*/CyJ mice, along with FACS-sorted CD8^+^ or CD4^+^ T cells from B6.SJL and *Itk^-/-^* mice, mixed at a 1:1 (WT:*Itk^-/-^*) ratio. Seven days post-transplantation, the mice were sacrificed and lymphocytes from the liver, small intestine, spleen, and skin-draining lymph nodes were isolated. Livers were perfused with PBS, dissociated, and filtered with a 70 μm filter. The small intestines were washed in media, shaken in strip buffer at 37°C for 30 min to remove the epithelial cells, and then washed, before digesting with collagenase D (100 mg/ml) and DNase (1 mg/ml) for 30 min in 37°C, and followed by filtering with a 70 μm filter. Lymphocytes from the liver and intestines were further enriched using a 40% Percoll gradient. The cells were analyzed for H2K^b^, CD45.1^+^ and CD45.2^+^ CD8^+^ T cells by flow cytometry, but we excluded any bone marrow-derived T cells (Thy1.1^+^).

### RNA Sequencing

T cells from WT C57Bl/6 or *Itk^-/-^* mice were MACS purified and FACS sorted, and 2 × 10^6^ FACS sorted CD8^+^ T cells were transplanted into BALB/c mice, along with _TCD_BM as described above. Seven days post transplantation, donor cells were purified from spleen. Samples were submitted to SUNY Upstate Medical University Sequencing core facility for RNA sequencing. We were unable to sort enough donor T cells from small intestine of the recipient mice that received *Itk^-/-^* T cells. Therefore, we generated RNA sequencing data from five groups: WT-Pre and *Itk^-/–^*Pre cells prior to transplantation; WT-Spleen, and *Itk^-/-^*Spleen using cells isolated from 7 days post-transplantation. Copy numbers were further analyzed in Gene Spring for normalization, quality control, correlation, principal component analysis, and gene differential expression. The sequencing data is deposited in (https://www.ncbi.nlm.nih.gov/geo/).

### Western Blotting

Cells were lysed in freshly prepared lysis buffer using RIPA buffer (Fisher Scientific) and Complete Protease Inhibitor Cocktail (Fisher Scientific) and centrifuged for 10 min at 4°C. Aliquots containing 70 μg of protein were separated on a 12–18% denaturing polyacrylamide gel and transferred to nitrocellulose membranes for immunoblot analysis using specific Abs.

### qPCR Assay

To confirm the differences observed in RNA sequencing, pre- and post-transplanted donor T cells were FACS sorted from recipient mice on H2K^b^ markers, and total RNA was isolated from T cells using the RNeasy kit from Qiagen (Germantown, MD). cDNA was made from total RNA using a cDNA synthesis kit (Invitrogen). qRT-PCR assay was performed with a premade customized plate (Fisher Scientific, Hampton, NH).

### Human Patient Samples

We also isolated plasma from GVHD patients and healthy donors and performed cytokine ELISAs on these plasma samples using multiplex ELISA kits (Biolegend, San Diego, CA). This work was done under approved IRB protocol 1522145-2.

### Statistics

All numerical data are reported as means with standard deviation. Data were analyzed for significance with GraphPad Prism. Differences were determined using one-way or two-way ANOVA and Tukey’s multiple comparisons tests, or with a student’s t-test when necessary. P-values less than or equal to 0.05 are considered significant. All transplant experiments are done with N=5 mice per group, and repeated at least twice, according to power analyses. Mice are sex-matched, and age-matched as closely as possible.

## Results

### Ablation of ITK Retains GVL effect but Avoids GVHD During Allo-HSCT

**Figure 1:**
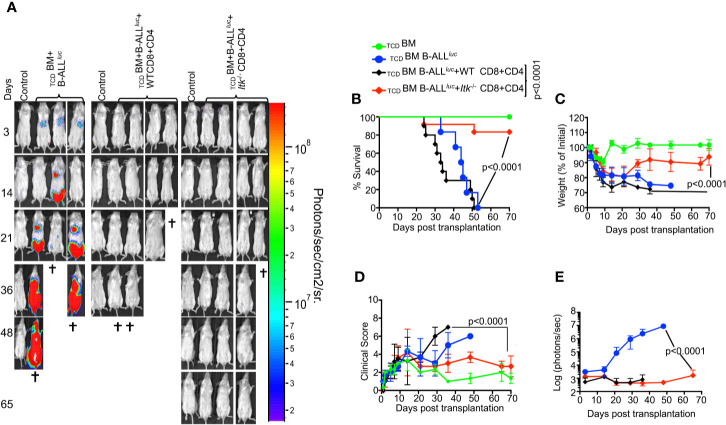
Absence of ITK avoids GVHD while retaining GVL effects during allo-HSCT. 1 × 10^6^ purified CD4+ and 1 × 10^6^ CD8+ T cells (WT or *Itk^-/-^)* were mixed at a 1:1 ratio, and transplanted along with 2 × 10^5^ B-ALL-*luc* cells into irradiated BALB/c mice. Host BALB/c mice were imaged using the IVIS imaging system 3 times a week. Group 1 received 10 × 10^6^ T cell depleted bone marrow only (labeled as _TCD_BM). Group 2 received 10 × 10^6^_TCD_BM along with 2 × 10^5^ B-ALL-*luc* cells (_TCD_BM+B-ALL *^luc^*+), Group 3 was transplanted with 1 × 10^6^ purified WT CD8^+^ and 1 × 10^6^ CD4^+^ T cells (1:1 ratio) along with 2 × 10^5^ B-ALL-*luc*+ cells 10 × 10^6^ (_TCD_BM+B-ALL *^luc^*+ WT CD8+CD4). Group 4 received 1 × 10^6^ purified *Itk^-/-^* CD8^+^ and 1 × 10^6^ CD4^+^ T cells (1:1 ratio) along with 2 × 10^5^ B-ALL-*luc*+ cells 10 × 10^6^ (_TCD_BM+B-ALL*^luc^*+ *Itk^-/-^* CD8+CD4). **(A)** Recipient BALB/c mice were imaged using IVIS 3 times a week. **(B)** The mice were monitored for survival, **(C)** changes in body weight, and **(D)** clinical score for 65 days post BMT. **(E)** Quantified luciferase bioluminescence of leukemia cell growth. Statistical analysis for survival and clinical score was performed using log-rank test and two-way ANOVA, respectively. For weight changes and clinical score, one representative of 2 independent experiments is shown (n = 3 mice/group for BM alone; n = 5 experimental mice/group for all three groups. Survival is a combination of 2 experiments. P values presented with each group. Two-way ANOVA and students t-test were used for statistical analysis. *Note:* *Controls are naïve mice used as negative control for* *bioluminescence* *(BLI)*.

To determine whether TCR-mediated activation of ITK impacts GVHD pathogenesis after allo-HSCT, we examined the effects of ITK signaling on donor CD4^+^ and CD8^+^ T cells in an allo-transplant model, using C57Bl/6 mice (MHC haplotype b) as donors and BALB/c mice (MHC haplotype d) as recipients. To induce GVHD, we used MHC-mismatched donors and recipients, _TCD_BM from B6.PL-*Thy1^a^*/CyJ (Thy1.1) mice, and T cells from C57BL/6 (B6) WT or *Itk^-/-^* mice. Lethally irradiated BALB/c mice were injected intravenously with 10 × 10^6^ wild-type (WT) _TCD_BM cells along with 2 × 10^6^ FACS-sorted donor T cells (1 × 10^6^ CD8^+^ and 1 × 10^6^ CD4^+^), followed by intravenous challenge with 2 × 10^5^ luciferase-expressing B-ALL-*luc* blast cells as described (16). Recipient BALB/c mice were monitored for cancer cell growth using IVIS bioluminescence imaging for over 60 days >>(**Figure 1A**). While leukemia cell growth was observed in T cell-depleted BM-transplanted mice without T cells, leukemia cell growth was not seen in mice transplanted with T cells from either WT or *Itk^-/-^* mice. As expected, mice transplanted with WT T cells cleared the leukemia cells but suffered from GVHD. In contrast, mice transplanted with *Itk^-/-^* T cells cleared the leukemia cells and displayed minimal signs of GVHD. Most animals transplanted with *Itk^-/-^* T cells survived for more than 65 days post-allo-HSCT (**Figure 1B**), with significantly better survival and reduced clinical scores compared to those transplanted with WT T cells [scored based on weight, posture, activity, fur texture, and skin integrity as previously described (19) (**Figures 1C, D**)]. BALB/c mice transplanted with *Itk^-/-^* T cells showed only residual tumor cell growth (as measured by bioluminescence), showing that the donor cells maintained GVT functions similar to WT T cells (**Figure 1E**). Donor CD8^+^ T cells are more potent than CD4^+^ T cells in mediating GVL effects, but both CD4^+^ and CD8^+^ T cells mediate severe GVHD in mice and humans (23–25). To determine whether CD4^+^ T cell-intrinsic ITK signaling might be sufficient to induce GVHD, we repeated the same experiments using purified CD4^+^ T cells from either WT or *Itk^-/-^* mice in the MHC-mismatch mouse model of allo-HSCT (B6→BALB/c) (**Supplementary Figures 1A–C**). Recipients of WT CD4^+^ T cells exhibited worse survival compared to mice receiving _TCD_BM cells alone (**Supplementary Figure 1A**). In contrast, recipients of _TCD_BM mixed with *Itk^-/-^* CD4^+^ T cells had greatly reduced mortality and clinical scores (**Supplementary Figure 1B**), indicating that CD4^+^ T cell-intrinsic ITK signaling can contribute to the severity of GVHD. Our results indicate that ITK signaling is dispensable for anti-leukemia immunity, but required for GVHD.

### T Cells Innate Memory Phenotype Is Not Sufficient for GVHD Effects, and the Regulatory Function of ITK in GVHD Is T Cell-Intrinsic

**Figure 2:**
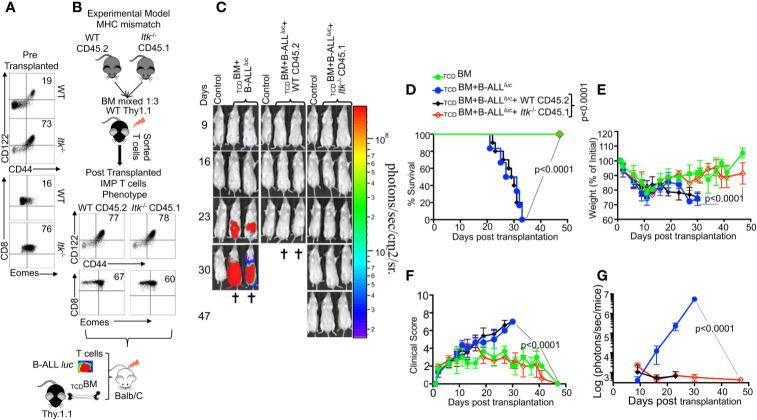
The regulatory function of ITK in GVHD is T cell-intrinsic. **(A)** Purified WT and *Itk^-/-^* CD8^+^ T cells were examined for expression of CD44, CD122, and Eomes prior to transplantation. **(B)** Whole bone marrow cells isolated from C57Bl/6 WT (CD45.2) and *Itk^-/-^* (CD45.1) mice were mixed in 1:3 ration WT: *Itk ^-/-^*, and transplanted into irradiated Thy1.1 C57Bl/6 mice. 9-10 weeks later CD8^+^ T cells were sorted by CD45.2 and CD45.1 expression (donor T cells) and exclusion of Thy1.1 positive (host T cells). Isolated sorted T cells were examined for expression of CD44, CD122, and Eomes and transplanted into irradiated BALB/c mice. This experiment was repeated more than three times. **(C)** Irradiated BALB/c mice were divided in four different groups and transplanted with the sorted T cells described in **(B)** as follows: Group one was transplanted with 10 × 10^6^_TCD_BM alone (_TCD_BM). Group two was transplanted with 10 × 10^6^_TCD_BM and 2 × 10^5^ B-ALL-*luc*, (_TCD_BM+B-ALL^luc^). Group three was transplanted with 10 × 10^6^_TCD_BM along with 1 × 10^6^ purified WT CD8^+^ T cells and 2 × 10^5^ B-ALL-*luc* (_TCD_BM+B-ALL*^luc^*+WT CD45.2). The fourth group was transplanted 10 × 10^6^_TCD_BM along with and 1 × 10^6^ purified *Itk^-/-^* CD8^+^ T cells and 2 × 10^5^ B-ALL-*luc* (_TCD_BM+B-ALL*^luc^*+*Itk^-/-^* CD45.1). These mice were monitored for leukemia cell growth using the IVIS system. **(D)** The mice were monitored for survival, **(E)** changes in body weight, and **(F)** clinical score for 47 days post BMT. For body weight changes and clinical score, one representative of 2 independent experiments is shown (n = 3 mice/group for BM alone; n = 5 experimental mice/group for all three groups). **(G)** Quantified luciferase bioluminescence of luciferase expressing B-ALL-*luc* cells. Statistical analysis for survival and clinical score was performed using log-rank test and two-way ANOVA, respectively. One representative experiment out of 2. Survival is a combination of 2 experiments, 3 mice per group of control _TCD_BM, and 5 mice per group for all of the experimental groups. P value presented with each figure. *Note:* *Controls are naïve mice used as negative control for BLI*.

The innate memory phenotype (IMP: CD44^hi^CD122^hi^ and Eomes^hi^) (26) of *Itk^-/-^* CD8^+^ T cells arises in the thymus during development, as opposed to memory CD8^+^ T cells that are also CD44^hi^, but largely arise in the periphery of WT mice in response to foreign antigens or due to homeostatic proliferation (27). We examined pre-transplanted CD8^+^ T cells for CD44^hi^CD122^hi^ and Eomes^hi^ expression, and observed that *Itk^-/-^* T cells expressed higher levels of CD44^hi^CD122^hi^ and Eomes^hi^ compared to CD8^+^ T cells from WT mice (**Figure 2A**). We sought to understand whether the emergence of IMP T cells is sufficient to separate GVHD from GVL. To test this, we generated WT IMP T cells using a mixed-bone marrow approach in which T cell-depleted BM from WT and *Itk^-/-^* mice were mixed at a 3:1 (WT: *Itk^-/-^*) ratio (26). The irradiated congenic (B6) Thy1.1 hosts were reconstituted with this mixture of _TCD_BM CD45.2^+^ WT and CD45.1^+^ *Itk^-/-^* BM cells, along with a control group receiving mixed CD45.2^+^ WT and CD45.1^+^ WT BM cells (**Figure 2B**). WT BM-derived CD8^+^ thymocytes that develop in such mixed BM chimera acquire an IMP phenotype due to their development in the same thymus as the *Itk^-/-^* T cells (26), which we also observed in our experiments (**Figure 2B**). Ten weeks after reconstitution of the T cell compartment, T cells derived from WT (CD45.2^+^Thy1.1^-^) and *Itk^-/-^* (CD45.1^+^) donor cells were sorted from the bone marrow chimeras. These sorted T cells were transplanted into irradiated BALB/c mice along with _TCD_BM in the allo-HSCT model as described above, and tested for their function in GVHD and GVL. Analysis of the BALB/c recipients of these different IMP CD8^+^ T cells indicates that WT IMP cells were not able to separate GVL and GVHD (**Figures 2C–G**). Thus, the appearance of IMP is not sufficient to separate GVHD from GVL.

**Figure 3:**
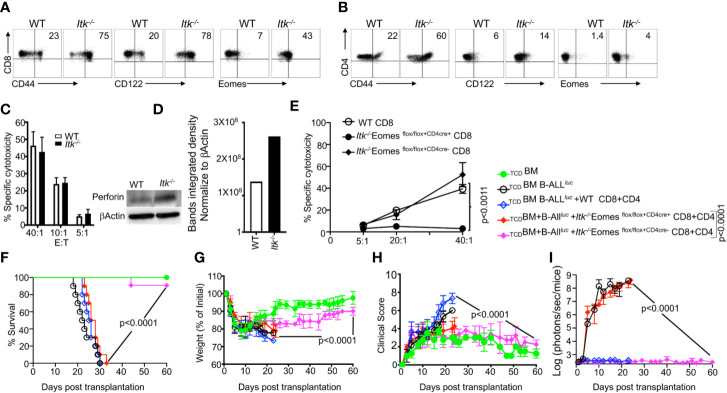
IMP T cells are not sufficient for GVL effect. **(A, B)** Purified WT and *Itk^-/-^* CD8^+^ and CD4^+^ T cells were examined for expression of CD44, CD122, and Eomes by flow cytometry. **(C)** Purified WT or *Itk^-/-^* T cells were transplanted into irradiated BALB/c mice, at day 7 purified T cells were sorted using H2K^d^, CD45.1 and CD45.2 expression. *Ex vivo* purified CD8^+^T cells were used in cytotoxicity assay against primary leukemia target B-ALL*luc*+ cells at a 40:1, 20:1, or 5:1 ratio. **(D)** Purified T cells were examined for perforin by western blot. Quantitative analysis of perforin expression by western blot with data normalized against β–Actin. **(E)** Purified WT or *Itk/Eomes* DKO donor T cells were transplanted into irradiated BALB/c mice. On day 7 donor T cells were purified as described and used in an *ex vivo* cytotoxicity assay against B-ALL*^luc^*-cells at 5:1, 20:1, and 40:1 ratio. **(F)** 1 × 10^6^ purified WT and *Itk^-/-^ Itk/Eomes* DKO CD8^+^ T cells and 1 × 10^6^ purified CD4^+^ T total of 2 × 10^6^ mixed CD4^+^ and CD8^+^ T cells were mixed and transplanted along with 2 × 10^5^ B-ALL-*luc* cells and 10 × 10^6^_TCD_BM into irradiated BALB/c mice. Host BALB/c mice were imaged using IVIS 3 times a week. Group one received 10 × 10^6^_TCD_BM alone as (_TCD_BM). Group two received 10 × 10^6^_TCD_BM along with 2 × 10^5^ B-ALL-*luc* cells (_TCD_BM+B-ALL*^luc^*). Group three were transplanted with 10 × 10^6^_TCD_BM and 1 × 10^6^ purified WT CD8^+^ T cells +1 × 10^6^ CD4^+^ T cells and 2 × 10^5^ B-ALL-*luc* cells (_TCD_BM+B-ALL*^luc^+*WT CD8+CD4). Group four received 10 × 10^6^ T _TCD_BM and 1 × 10^6^ purified CD8^+^ T cells +1 × 10^6^ CD4^+^ T cells from *Itk/Eomes* DKO along with 2 × 10^5^ B-ALL-*luc* cells (_TCD_BM+B ALL*^luc^+ Itk^-/-^* EomesFF+CD4cre CD8+CD4). Group five received 10 × 10^6^_TCD_BM and 1 × 10^6^ CD8^+^ T cells +1 × 10^6^ CD4^+^ purified T cells *Itk/Eomes* DKO CD4^+^ T cells along with 2 × 10^5^ B-ALL-*luc* cells (_TCD_BM+B-ALL*^luc^*+ *Itk^-/-^* ^EomesFF+CD4cre-^ CD8+CD4). **(F)** The mice were monitored for survival, **(G)** body weight changes, and **(H)** clinical score for 60 days post BMT. For weight changes and clinical score, one representative of 2 independent experiments is shown (n = 3 mice/group for BM alone; n = 5 experimental mice/group for all three group. The survival groups are a combination of all experiments. **(I)** Quantitated luciferase bioluminescence of tumor growth. Statistical analysis for survival and clinical score was performed using log- Two-way ANOVA were used for statistical analysis confirming by students *t* test, p values are presented. *Note:* *Controls are naïve mice used as negative control for bioluminescence (BLI)*.

As previously discussed, *Itk^-/-^* CD8^+^ and CD4^+^ T cells exhibit attenuated TCR signaling and an IMP (26), as indicated by expression of high levels of CD44, CD122, and Eomes, specifically by CD8^+^ T cells (**Figures 3A, B**). To examine whether these IMP T cells from *Itk* ^-/-^ mice mount GVL responses, we utilized the MHC-mismatch mouse model of allo-HSCT (WT, *Itk^-/-^*→BALB/c, i.e., H2K^b+^→H2K^d+^). We then sorted H2K^b+^donor T cells back from recipient mice and determined their cytotoxicity against B-ALL-*luc* cells. We found that these donor cells effectively killed primary leukemia cells *in vitro*, even in the absence of ITK (**Figure 3C**). Moreover, we observed significantly increased expression of perforin in CD8^+^ T cells from *Itk^-/-^* mice compared to T cells from WT mice, in the absence of activation (**Figure 3D**). Our findings demonstrate that CD8^+^ T cells from *Itk^-/-^* mice have enhanced activation, and exert cytotoxicity against primary leukemia cells.

IL-4 is known to upregulate Eomes in CD8^+^ T cells (26, 28), which we verified by comparing T cells from WT and *Itk/Il4ra* double KO (DKO) mice. Removing IL-4 signaling from the *Itk^-/-^* mice led to decreased expression of Eomes in *Itk^-/-^* T cells compared to T cells from *Itk^-/-^* and WT pre-transplanted (**Supplementary Figure 2A**). Next, we used the short-term allo-HSCT model, where T cells from WT or *Itk/Il4ra* DKO were transplanted into irradiated BALB/c mice. 7 days post transplantation, WT or *Itk/Il4ra* DKO donor T cells were then sorted back from the BALB/c recipient mice, and Eomes expression on these donor T cells was determined. We did not observe any differences between the donor WT or *Itk/Il4ra* T cells upon allo activation (**Supplementary Figures 2A–C**). Next, we tested the function of *Itk/Il4ra* DKO T cells in the long term allo-HSCT model, and observed that donor T cells from *Itk/Il4ra* DKO mice did not induce GVHD, and most of the animals survived compared to recipients of WT T cells (**Supplementary Figure 2D**). BALB/c transplanted with *Itk/Il4ra* donor T cells also had much less weight loss and significantly better clinical scores compared to BALB/c mice transplanted with WT donor T cells (**Supplementary Figures 2D–G**). Furthermore, *Itk/Il4ra* DKO donor T cells cleared leukemia cells without inducing GVHD. These data show that the IMP T cell phenotype may not be critical for GVHD, but modulating ITK does impact GVHD without affecting GVL.

To investigate the role of Eomes in clearing leukemia cells and in cytotoxic function, we crossed *Itk^-/-^* mice with *Eomes* ^flox/flox^ mice, and crossed these offspring with CD4cre mice, to delete Eomes specifically in T cells (28, 29) to generate (*Itk/Eomes* DKO). We performed similar allo-HSCT experiments as described above, and used WT or *Itk/Eomes* DKO T cells. Seven days post-transplant, donor T cells were sorted using H2K^b^ expression, and *in vitro* cytotoxicity assays were performed at a 5:1, 20:1 and 40:1 ratio (effector: target). Our data show that *ex vivo* donor *Itk/Eomes* DKO were unable to kill cancer targets (**Figure 3E**). To examine the role of Eomes in the allo-HSCT model, BALB/c mice were lethally irradiated and injected intravenously with 10 × 10^6^ WT _TCD_BM cells along with FACS-sorted CD8^+^ and CD4^+^ T cells from donor mice (WT, *Itk^-/-^Eomes* DKO). This was followed by intravenous challenge with 2 × 10^5^ luciferase-expressing B-ALL-*luc* blast cells as described (17). Recipient animals transplanted with WT T cells cleared the tumor cells but had reduced survival and GVHD (**Figures 3F–I**). Recipient animals transplanted with *Itk^-/-^Eomes* DKO T cells however, did not cleared the leukemia cells without showing signs of GVHD (**Figure 3I**). Notably, recipient animals transplanted with *Itk/Eomes* DKO T cells mice were unable to clear the tumor and all died from cancer burden. These data provided further evidence that Eomes is required for the GVL effect.

### ITK Deficiency Results in Reduced Cytokine Production

**Figure 4:**
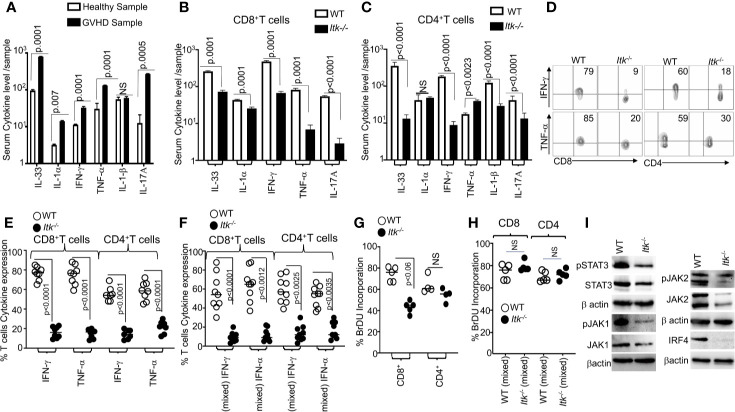
ITK deficiency results in reduced cytokine production. **(A)** Serum from several GVHD patients was isolated and examined for inflammatory cytokine production (IL-33, IL1α, IFN-γ and TNF-α, IL1β and IL-17A) as determined by ELISA. **(B**, **C)** 1 × 10^6^ purified WT or *Itk^-/-^* CD8^+^ T or CD4^+^ T cells were separately transplanted with into irradiated BALB/c mice. At day 7 post allo-HSCT, recipient BALB/c were euthanized and serum cytokines (IL-33, IL1α, IFN-γ, and TNF-α and IL-17A) were measured by ELISA. **(D)** Intracellular IFN-γ and TNF-α expression by donor CD8^+^ and CD4^+^ T cells after stimulation with anti-CD3/anti-CD28 as determined by flow cytometry. **(E**, **F)** Combined data from 3 independent experiments is shown for each experiment shown in figures. **(F)** Flow cytometry analysis of purified WT and *Itk^-/-^* T cells that were mixed at a 1:1 ratio for transplantation into irradiated BALB/c mice. At day 7 donor T cells were gated for expression of H-2K^b^, CD45.1, and CD45.2 and intracellular expression of IFN-γ and TNF-α was analyzed by flow cytometry after stimulation with anti-CD3/anti-CD28. Combined data from four independent experiments is shown, and the p value for each experiment is shown. **(G)** Purified WT or *Itk^-/-^* donor CD8^+^ and CD4^+^ T cells were transplanted into irradiated BALB/c. At day 7 donor cells were analyzed for donor T cell proliferation by examining BrdU incorporation by flow cytometry. **(H)** Purified WT and *Itk^-/-^* donor T cells were mixed at a 1:1 WT: *Itk^-/-^* ratio and transplanted into irradiated BALB/c mice, at day 7 splenic donor T cells were gated for the expression of H-2K^b^, CD45.1, and CD45.2 and analyzed for BrDU incorporation. **(I)** Purified WT and *Itk^-/-^*T cells were stimulated with CD3 and CD28 overnight examined for the expression and phosphorylation of IRF4, JAK1/2 and STAT3 by western blot. For statistical analysis we used two-way ANOVA and student’s *t* test, p values are presented.

It is known that the conditioning regimen for allo-HSCT elicits an increase in the production of inflammatory cytokines by donor T cells, and this is considered to be one of the hallmarks of GVHD pathogenesis (30). We obtained blood samples from GVHD patients and healthy donors and examined the levels of serum inflammatory cytokines such as IL-33, IL-1α, IFNγ, TNFα and IL-17A. We observed that patients with GVHD have significantly higher levels of serum proinflammatory cytokines compared to healthy controls (**Figure 4A**). Next, we assessed cytokine production by *Itk^-/-^* CD8 and CD4 T cells in our allo-HSCT model (B6→BALB/c), examining the levels of serum inflammatory cytokines such as IL-33, IL-1α, IFN-γ, TNF-α and IL-17A on day 7 post allotransplantation (**Figures 4B, C**). We found that serum IFN-γ and TNF-α were significantly reduced in recipients that received *Itk^-/-^* CD8^+^ T or CD4^+^ T cells compared to those that received WT CD8^+^ or CD4^+^ T cells (**Figure 4B, C**). Thus, we confirmed that the findings in our pre-clinical model correlated with what we found in human GVHD samples. We also isolated *Itk^-/-^* donor T cells from the secondary lymphoid organs of recipients using anti-H2K^b^ antibodies (expressed by donor C57Bl/6 cells). 7 days post allo-transplantation, cells were stimulated with anti-CD3/CD28 (**Figure 4D**), or PMA/ionomycin [to bypass the proximal TCR signaling defect (31) (**Supplementary Figure 3**)], in the presence of Brefeldin A, or left unstimulated for 6 h, followed by analysis of IFN-γ and TNF-α cytokine production. *Itk^-/-^* T cells were capable of producing IFN-γ and TNF-α at levels comparable to WT cells when both CD8^+^ and CD4^+^ T cell signaling was bypassed by re-stimulation with PMA and ionomycin (**Supplementary Figure 3**). However, the *Itk^-/-^* cells produced significantly less inflammatory cytokines when stimulated *via* TCR/CD28 than WT cells did (**Figures 4D, E**). Next, we determined whether the reduction of cytokine production by *Itk^-/-^* donor T cells was due to cell-intrinsic or -extrinsic factors. We mixed purified *Itk^-/-^* CD8^+^ T and CD4 T cells with purified WT CD8^+^ or CD4^+^ T cells separately at a 1:1 ratio, and transplanted the mixed cells into irradiated BALB/c mice as described above. On day 7, donor T cells were isolated from recipient mice using H2K^b+^ and examined for IFN-γ and TNF-α expression as described above. We found that WT donor CD8^+^ and CD4^+^ T cells produced higher levels of inflammatory cytokines than *Itk^-/-^* donor CD8^+^ and CD4^+^ T cells, respectively, suggesting that the reduced cytokine production observed by *Itk^-/-^* donor T cells is T cell-intrinsic (**Figure 4F**).

We next examined donor CD4^+^ and CD8^+^ T cell proliferation using a BrdU incorporation assay. 7 days post allo-transplantation as described above, transplanted splenic CD4^+^ and CD8^+^ T cells were examined for proliferation by BrdU incorporation. *Itk^-/-^* donor CD8^+^ showed statistically significantly reduced proliferation compared to WT donor CD8^+^ T cells, although there was no difference in proliferation between WT and *Itk^-/-^* CD4^+^ T cells (**Figure 4G**). To determine if the reduced proliferation of *Itk^-/-^* donor T cells was due to cell-intrinsic mechanisms, we mixed sort purified mixed *Itk^-/-^* and WT CD4^+^ or *Itk^-/-^* and WT CD8^+^ at a 1:1 ratio, followed by transplantation as described above. Interestingly, no difference was observed in BrdU incorporation in donor T cells from spleens of recipient mice between WT and *Itk* ^-/-^ donor CD4^+^ and CD8^+^ T cells in the mixed transplant models, indicating that the reduced proliferation of donor *Itk^-/-^* T cells proliferation was due to cell-extrinsic effects (**Figure 4H**). Thus, both cell intrinsic and extrinsic mechanisms regulate the behavior of *Itk* ^-/-^ CD8^+^ and CD4^+^ donor T cells.

The transcription factor IRF4 has been shown to play critical roles in maintaining TCR signaling, including TCR signal strength such as those regulated by ITK (32). The JAK/STAT signaling pathway is also critical for the response of T cells to cytokines (33, 34). To examine whether there was a difference in these signaling pathways between WT and *Itk^-/-^* donor T cells in the GVHD and GVL model, we examined expression of IRF4, JAK1, JAK2 and STAT3 by purified splenic T cells that had been stimulated overnight with CD3 and CD28 followed by lysis for analysis of protein. Our data showed that *Itk^-/-^* donor T cells expressed significantly less IRF4, JAK1, JAK2, and STAT3 as well as phosphorylated forms of JAK1, JAK2 and STAT3 (**Figure 4I** and **Supplementary Figures 4A–D**). Our data suggest that the lack of ITK affects the expression of IRF4, and thus the amount of cytokine signals the cells received. These data may explain the reduced cytokine production and proliferation in *Itk^-/-^* T cells observed above.

### ITK Differentially Regulates Gene Expression in T Cells During GVHD

**Figure 5:**
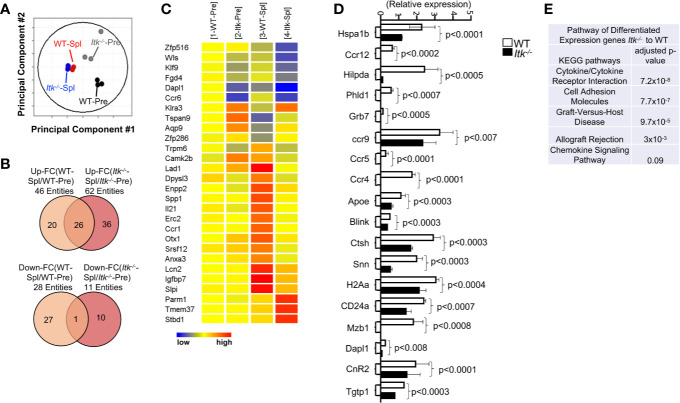
ITK differentially regulates gene expression in T cells during GVHD. WT and *Itk^-/-^* CD8^+^ T cells were FACS sorted then transplanted into irradiated BALB/c mice. At day 7 post-transplant, donor T cells were sort-isolated (based on expression of H-2K^b^, CD3 and CD8) from host spleen. Sorted donor T cells were subjected to RNA sequencing. **(A)** Principal component analysis of genes with ≥2-fold change in any pairs of group combinations, with false discovery rate (FDR) ≤ 0.05. WT-Pre and *Itk^-/–^*Pre denotes cells prior to transfer, and WT-Spl, and *Itk^-/–^*Spl denotes cells isolated from the spleen (Spl) of the recipients post-transfer. **(B)** Venn diagram of genes that are ≥2-fold up- or down- regulated in the indicated comparisons, with FDR (*P*) ≤ 0.05. **(C)** Heat map of differentially expressed genes listed as (1) WT pre. (2) ITK-/- pre, (3) WT post spleen, and (4) ITK-/- post spleen. **(E)** Differentially expressed genes were enriched for pathway analysis comparing WT and *Itk^-/-^.* **(D)** WT and *Itk^-/-^* CD8^+^ T cells were FACS sorted then transplanted into irradiated BALB/c mice. At day 7 post-transplant, donor T cells were sort-isolated (based on expression of H-2K^b^, CD3 and CD8) from host spleen and small intestine (Gut). Total RNA was isolated from sorted donor T cells were and qPCR was performed.

**Figure 6:**
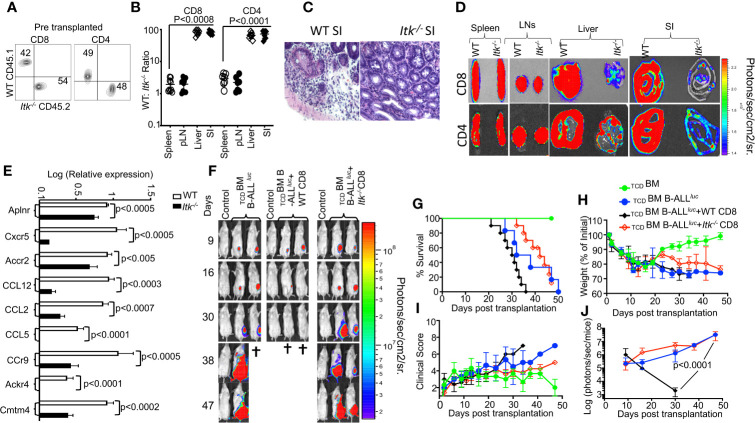
ITK signaling is required for T cell migration to the GVHD target tissues. **(A)** Irradiated BALB/c mice were allo-HSCT-transplanted and injected with FACS-sorted WT and *Itk^-/-^* CD8^+^ T and CD4^+^ T cells mixed at a 1:1 ratio. FACS analysis of sorted T cells pre-transplant shown. **(B)** At day 7 post-BMT, the spleen, liver, and small intestine (SI) were examined for donor WT and *Itk^-/-^* T cells. The ratio of WT: *Itk^-/-^* CD8^+^ and CD4^+^ T cells in the organs was determined. **(C)** At day 7 post-allo-HSCT, small intestines were examined by H&E staining. **(D)** Irradiated BALB/c mice were BM-transplanted and injected with CD8^+^ T CD4^+^ T cells from luciferase-expressing WT or *Itk^-/-^* mice. **(E)** On Day 7 post-allo-HSCT, donor T cells were isolated and examined for the expression of Aplnr, Cxcr5, Accr2, CCL12, CCL2, CCL5, CCr9, Ackr4, and Cmtm4 using q-RTPCR. P values were calculated using 2-way ANOVA and Student’s *t* test, p values are listed. **(F)** Irradiated BALB/c mice were transplanted with C57Bl/6-derived BM and FACS-sorted WT or *Itk^-/-^* 1 × 10^6^ CD8^+^ T cells, and challenged subcutaneously with 2 × 10^5^luciferase-expressing B-All *luc* cells. Recipient animals were monitored for weight changes. Group one of recipient mice was transplanted with 10 × 10^6^_TCD_ BM. The second group of recipient mice was transplanted with 10 × 10^6^_TCD_BM and 2 × 10^5^ primary B-ALL luc+ cells(_TCD_BM+B-ALL*^luc^*). The third group of recipient mice was transplanted with 10 × 10^6^_TCD_BM along with 1 × 10^6^ T cell from WT mice along with 2 × 10^5^ B-ALL-*luc+* cells (_TCD_BM+B-ALL*^luc^*+WT CD8). The fourth group of recipient mice was transplanted with 10 × 10^6^_TCD_BM and 1 × 10^6^ T cell from *Itk* ^-/-^ mice along with 2 × 10^5^ B-ALL-*luc+* cells. (_TCD_BM+B-ALL*^luc^*+ *Itk* ^-/-^ CD8). Representative bioluminescence images of leukemia cell-bearing mice on days 9, 16, 30, 38, and 47 are shown. *Note:* *Controls are naïve mice used as negative control for* bioluminescence *(BLI).* **(G)** Animals were monitored for survival over 47 days, **(H)** for changes in weight loss, **(I)** and for clinical score. **(J)** Recipient mice were monitored for leukemia cell growth using the IVIS imaging system and quantified data is shown. For weight changes and leukemia cell growth, one representative of 2 independent experiments is shown (n = 3 mice/group for control, n = 5 mice for WT, and n = 5 mice for *Itk^-/-^).* Survival groups were combined from both experiments. P values were calculated using two-way ANOVA and Student’s *t* test, p values are listed.

As an unbiased approach to further explore differences between WT and *Itk^-/-^* CD8^+^ T cells, we employed RNA sequencing analysis to examine the differences in gene expression between WT and *Itk^-/-^* CD8^+^ T cells following allo-HSCT. We sort-purified donor WT and *Itk^-/-^* CD8^+^ T cells (using H-2K^b^ antigen expressed by donor T cells) before and 7 days after they were transferred into irradiated BALB/c recipients, and RNA sequences was done. Although WT and *Itk^-/-^* CD8^+^ T cells are distinct prior to transplantation due to the enhanced IMP in the absence of ITK, WT and *Itk^-/-^* T cells which homed to the spleen post-transplantation are similar as revealed by the fact that they clustered within a close proximity in the Principal Component Analyses (PCA) (**Figure 5A**). We were unable to collect enough cells from the intestine of the *Itk^-/-^* T cell recipients, since they are deficient in homing to the intestine (see **Figures 6B–D**). To further determine the differentially expressed genes that are unique in WT CD8^+^ T cells and associated with their ability to home to the GVHD target organs, we compared the lists of genes that were up- or down- regulated after the cells were transferred into the recipients and homed to different organs. Genes that are differentially expressed in WT T cells that were able to home to the GVHD target organ may reveal signals that are deficient due to the absence of ITK. We therefore extracted the list of genes that are up- or down-regulated in only WT T cells isolated from the gut of the recipient’s post-transplantation (**Figures 5B, C** shows 20 up-regulated and 27 down-regulated genes). The differentially expressed genes between WT and *Itk* ^-/-^ donor T cells were enriched for transcripts encoding lymphocyte homing molecules such as adhesion molecules and chemokine signaling proteins, which might contribute to the defective homing capability of *Itk* ^-/-^ donor T cells (**Figure 5E**). The results of critical genes that were differentially expressed were confirmed by q-RT-PCR (**Figure 5D**). Using pathway enrichment analyses, our data also revealed a critical role for ITK in regulating genes involved in T cell cytokine/cytokine receptor interaction, cell adhesion, graft-versus-host disease, allograft rejection, and chemokine signaling pathways (**Figure 5E**). These data suggest that ITK regulates the expression of signature genes associated with the homing of the transplanted cells into the GVHD targeted organs, while it does not have an apparent effect on T cell homing in the spleen. This may, in part, explain the ability of *Itk^-/-^* T cells to maintain GVL effects while being unable to home to the GVHD target organs and participate in GVHD.

### ITK Signaling Is Required for T Cell Migration to the GVHD Target Tissues

GVHD involves early migration of alloreactive T cells into the target organs, followed by T cell expansion and tissue destruction. Modulation of alloreactive T cell trafficking has been suggested to play a significant role in ameliorating experimental GVHD (35). Therefore, we examined the trafficking of donor T cells to GVHD target tissues as previously described (35). Irradiated BALB/c recipient mice were injected with CD8^+^ and CD4^+^ T cells from *Itk^-/-^* (CD45.2^+^) and WT B6LY5(CD45.1^+^) mice mixed at a 1:1 ratio (**Figure 6A**), and at 7 days post transplantation, recipient mice were examined for the presence of donor CD8^+^ and CD4^+^ T cells in the spleen, lymph nodes, liver and the small intestines. While the WT: *Itk^-/-^* CD8^+^ and CD4^+^ T cell ratio remained approximately 1:1 in the spleen and lymph nodes (**Figure 6B**), this ratio in the liver and small intestine was significantly elevated, suggesting that *Itk^-/-^* CD8^+^ and CD4^+^ T cells were defective in migration to and/or expansion in those tissues. Using histological staining for H&E, we also observed significant leukocyte infiltration into GVHD target organs – liver, skin, and small intestine (SI) (36) in WT T cell recipients but not in *Itk^-/-^* T cell recipients (**Figure 6C**). As an alternative approach, we tracked both CD8^+^ and CD4^+^ T cells in allo-BMT mice by using donor CD8^+^ and CD4^+^ T cells from WT and *Itk^-/-^* mice that also express luciferase, which could be monitored by bioluminescence (37). We observed that both CD8^+^ and CD4^+^ donor T cells from *Itk^-/-^* mice had significantly impaired residency in GVHD target organs, including the liver and small intestine (SI), compared to WT, despite no differences in spleen and lymph nodes (**Figure 6D**). Secondary lymphnodes (spleen and Lymph nodes) and GVHD target oragns small intestine (SI), and liver were quantified luciferase bioluminescence (**Supplementary Figures 5A, B**). In the mixed T cell transfer model, we had determined that *Itk^-/-^* T cell proliferation was comparable to that of WT cells; therefore, it is very likely that the reduced number of *Itk^-/-^* T cells in the liver and small intestine was due to impaired T cell trafficking. Pro-inflammatory conditioning treatment may promote T cell migration into GVHD target tissues (38, 39). Indeed, in the same mixed T cell transfer model, we found that chemokine and chemokine receptor expression (Aplnr, Cxcr5, Accr2, CCL12, CCL2, CCL5, Ccr9, Ackr4, and Cmtm4) was also significantly reduced in *Itk^-/-^* CD8^+^ and CD4^+^ T cells at day 7 post-transplantation (**Figure 6E**). These data suggest that *Itk^-/^* ^-^ CD8^+^ T cells display attenuated chemokine receptor expression, which correlates with defective migration to GVHD target organs and reduced target organ pathology.

Given that *Itk^-/-^* T cells exhibit defective migration to target organs of GVHD, we predicted that although *Itk^-/-^* T cells can clear leukemia cells in the blood and secondary lymphoid organs, they would not be able to kill leukemia cells that reside in tissues. To test this possibility, lethally irradiated BALB/c mice were BM-transplanted together with FACS-sorted WT or *Itk^-/-^* CD8^+^ T cells, and challenged with subcutaneously injected B-All *luc* cells. Although *Itk^-/-^* CD8^+^ T cells did not cause GVHD, the subcutaneously injected leukemia cells were cleared only in mice transplanted with WT CD8^+^ T cells, and not in those given *Itk^-/-^* CD8^+^ T cells (**Figure 6F–J**). Together, these data suggest that the ITK signaling in T cells can separate GVHD from GVL effects, but only for leukemia cells that reside in the circulation and in secondary lymphoid organs (such as hematologic malignancies).

## Discussion

In this report, we demonstrate that the absence of the TCR-regulated kinase ITK significantly suppresses GVHD, while maintaining the GVL effect in models of allo-HSCT. Loss of ITK also altered expression of IRF4, and the JAK/STAT pathway components JAK1, JAK2, and STAT, which play critical roles in controlling cytokine expression (14, 39). Transcriptome analysis by RNA sequencing revealed that ITK signaling controls chemokine receptor expression during this process, which in turn affects the ability of donor T cells to migrate to GVHD target organs. Taken together, these data suggest that ITK could represent a potential target for the separation of GVHD and GVL responses after allo-HSCT.

The ability of *Itk^-/-^* T cells to induce GVL without causing GVHD indicates that the ITK signaling pathway is involved in the pathogenesis of GVHD. *Itk^-/-^* T cells develop into IMP cells (CD122^+^ CD44^hi^ phenotype) in the thymus, and it is possible that such cells are responsible for the GVHD and GVL effects we observe. In experiments where WT T cells developed into IMPs, we found that they retained the capacity to induce both acute GVHD and GVL, suggesting a T cell-intrinsic function of ITK in promoting GVHD during allo-HSCT. Similarly, the cytotoxicity of *Itk^-/-^* CD8^+^ T cells is not dependent on the IMP. While IMP cells express significantly higher Eomes compared to their WT non-IMP counterparts, we found that IMP CD8^+^ T cells are not responsible for distinguishing GVHD and GVL. To our surprise, we noted that *Itk^-/-^* CD8^+^ T cells exhibit similar or higher *in vitro* cytotoxicity compared to WT CD8^+^ T cells. This may be due to the higher levels of perforin expressed by *Itk^-/-^* T cells compared to WT T cells.

Our data also show that *Itk^-/-^* donor CD4^+^ and CD8^+^ T cells exhibit reduced expression of chemokine receptors compared to WT counterparts. Moreover, the migration of *Itk^-/-^* donor T cells to target organs was also severely defective, reflecting the reduced expression of key chemokine receptors. The defective migration of *Itk^-/-^* CD8^+^ and CD4^+^ T cells likely contributes to the attenuation of GVHD, since these T cells continue to display GVL effects against leukemia cells that were injected intravenously and reside in secondary lymphoid organs. In contrast, WT but not *Itk^-/-^* CD8^+^ T cells were able to inhibit leukemia cell growth when the leukemia cells were injected subcutaneously. The compartmentalization of T cells to secondary lymphoid organs can be an effective strategy for preventing GVHD, while leaving GVL effects against hematologic malignancies intact. It is noteworthy that Ibrutinib, an inhibitor of BTK which can also inhibits ITK, is able to reduce chronic GVHD (12). In addition, previously published work showed that IFN-γR signaling constitutes a major mechanism for donor T cell migration to GVHD target organs (40, 41), and we observed that the lack of ITK affects production of IFN-γ. The retention of T cells to secondary lymphoid organs by FTY720-mediated inhibition of S1P1 also ameliorates GVHD while maintaining GVL effects (42, 43). Similarly, inhibition of T cell migration to GVHD target organs by targeting the chemokine receptors CCR2 or CCR5 protects against GVHD-induced pathology (44, 45), which at least with CCR2 deficiency was shown to preserve the GVL effect. Importantly, in a clinical study, CCR5 blockade by a small molecule antagonist led to a reduction in GVHD with no significant difference in relapse rates, suggesting that blocking T cell migration to target tissues could reduce GVHD severity without compromising the beneficial GVL effect (45). In addition, the inhibition of CXCR3 ameliorates GVHD in allo-HSCT mice (46). Activated alloreactive CD8^+^ T cells upregulate the expression of CX3CR1 and CXCR6 after allo-HSCT (47, 48), and these receptors are important for the homing of CD8^+^ T cells to the liver and intestines. Thus, CXCR6 deficiency or blockade of the CXCR3 and CXCR6 ligands attenuates GVHD (47). Importantly, the GVL effect is still maintained under these conditions (49). Thus, blocking T cell migration by chemokine receptor blockade could be beneficial in the treatment of GVHD after allo-HSCT. Since activated *Itk^-/-^* T cells displayed significantly reduced expression of chemokine receptors, the compartmentalization of CD8^+^ T cells to secondary lymphoid organs likely contributes to the preservation of GVL effects while severely attenuating GVHD (48).

Although suppression of TCR signaling can prevent GVHD, the complete suppression of T cell responses negates the beneficial GVL effect that is also provided by the same donor T cells after allo-HSCT (50). The fact that mice transplanted with *Itk^-/-^* T cells are able to mount GVL responses is an exciting feature. The preservation of the GVL response could have occurred for several reasons. First, the proliferation and cytotoxic activity of *Itk^-/-^* T cells are preserved compared to pro-inflammatory cytokine production. The manifestations and severity of GVHD are highly influenced by local cytokines, which then activate transcription factors and drive development toward a cytokine storm. In addition, proinflammatory cytokines exert direct effects on GVHD target tissues (51–53). Indeed, the presence of cytokine storm is considered one of the hallmarks of GVHD pathogenesis (54), and our data showed that cytokine production was significantly reduced in mice that received *Itk^-/-^* T cells. We also confirmed that cytokine production is T cell-intrinsic while proliferation is T cell-extrinsic. To explore the potential mechanism of this observed difference in cytokine and chemokine receptor expression between WT and *Itk^-/-^* donor CD4^+^ and CD8^+^ T cells, we analyzed key transcription factors and pathways that may be involved in these processes. We found significant differences in expression of the transcription factor IRF4 and the JAK/STAT signaling pathways, which regulate the expression of key molecules required for the maintenance of T cell effector function, cytokine production, and chemokine receptor upregulation. Since IRF4 has been shown to play critical roles in modulating TCR signal strength and T cell function (32), it is likely that reduction in the activation of IRF4 and of the JAK/STAT pathway contribute to reduced cytokine expression, thus alleviating the cytokine storm in GVHD (15). Our data show that the reduced proliferation seen in donor T cells from *Itk^-/-^* mice is cell-extrinsic. ITK deficiency has been shown previously to affect T cell proliferation (54) and cytokine production, but during allogenic activation, ITK-deficient T cells can still proliferate. This might be due to the redundant function of ITK and other Tec kinases (55). This finding is in line with our cytokine data, which show that *Itk^-/-^* T cells produce less cytokines, both in serum and on a per-cell basis. When transplanting either CD4^+^ or CD8^+^ T cells in a 1:1 ratio of WT:*Itk^-/-^* cells, we observed similar levels of proliferation for both WT and *Itk^-/-^* donor cells. Our data therefore provide further evidence that donor T cell proliferation is influenced by inflammatory conditions (56).

All together our data show that attenuating TCR signaling reduces donor T cell-mediated cytokine production, resulting in less severe GVHD. In addition, the inability of T cells to migrate to target organs may also affect this process, and thus explains the reduced ability of the *Itk^-/-^* donor T cells to induce GVHD.

## Data Availability Statement

The RNAseq data submission has been approved by NCBI GEO under the accession reference GSE161160. All data will be available to anyone.

## Ethics Statement

The studies involving human participants were reviewed and approved by IRB net 1140566-4. The ethics committee waived the requirement of written informed consent for participation. The animal study was reviewed and approved by SUNY Upstate Medical University and Cornell University.

## Author Contributions

MM, WH, AS, QY, SD, AB, and MK performed experiments. WT, YC, JP, and TG provided valuable reagents. RH assisted with data analysis, experimental design, scientific discussion, and manuscript editing. WH, QY, AA, AB, and MK designed experiments, analyzed the data, and wrote the manuscript. All authors contributed to the article and approved the submitted version.

## Funding

National Blood Foundation Scholar Award to MK and the National Institutes of Health (NIH LRP #L6 MD0010106 and AI130182 to MK, AI120701 and AI126814 to AA, R35ES028244 to AA and Gary Perdew, AI129422 to AA and WH, and AI146226 and GM130555-sub6610 to WH).

## Conflict of Interest

AA receives research support from 3M Corporation. WH receives research support from Mega Robo Technologies.

The remaining authors declare that the research was conducted in the absence of any commercial or financial relationships that could be construed as a potential conflict of interest.

## Supplementary Material

Supplementary Figure 1
*Itk^-/-^* CD4^+^ T cells exhibit attenuated induction of GVHD compared to WT T cells. **(A)** 10 × 10^6^_TCD_BM and 1 × 10^6^ purified WT or *Itk^-/-^* CD4^+^ T cells were transplanted into irradiated BALB/c mice. **(A)** The mice were monitored for survival, **(B)** changes in body weight, and (**C**) clinical score for 70 days post-BMT. For weight changes and clinical score, one representative of 2 independent experiments is shown (n = 3 mice/group for BM alone; n = 5 experimental mice/group for all three groups). The p values are presented. Two-way ANOVA and Student’s *t* test were used for statistical analysis.

Supplementary Figure 2 IMP T cells are not sufficient for GVHD effect. **(A-C)** *Itk/Il4ra* DKO and WT T cells were examined for Eomes expression pre- and post-transplantation. **(D)** 2 × 10^6^ purified WT and *Itk/Il4ra* DKO CD8^+^ T and 1 × 10^6^ purified CD4^+^ T cells were mixed and transplanted along with 2 × 10^5^ primary B-ALL-*luc*+ cells into irradiated BALB/c mice. Recipient BALB/c mice were imaged using IVIS 3 times a week. Group one received 10 × 10^6^_TCD_BM alone (_TCD_BM). Group two received 10 × 10^6^_TCD_BM along with 2 × 10^5^ B-ALL-*luc* cells (_TCD_BM+B-ALL*^luc^*). Group three was transplanted 10 × 10^6^_TCD_BM with 2 × 10^6^ purified (CD8^+^ and CD4^+^) from WT mice and 2 × 10^5^ B-ALL-*luc* cells (_TCD_BM+B-ALL *^luc^* *+*WT CD8+CD4). Group four was transplanted 10 × 10^6^_TCD_BM and 2 × 10^6^ purified T cells (CD8^+^ and CD4^+^) from *Itk/Il4ra* DKO along with 2 × 10^5^ B-ALL-*luc* cells (_TCD_BM+B-ALL*^luc^*+ *Itk/Il4ra* DKO CD8^+^CD4). Recipient animals were monitored for survival, **(E)** changes in weight, and **(F)** clinical score. **(G)** Leukemia cell growth was monitored as in **Figure 1**, and quantitated bioluminescence is shown. One representative of 2 independent experiments is shown (n = 3 mice/group for BM alone; n = 5 experimental mice/group for all three groups. The survival groups were combinations of all experiments.

Supplementary Figure 3
*Itk^-/-^* T cells are capable of cytokine production. Purified WT and *Itk^-/-^* T cells were transplanted into irradiated BALB/c mice. At day 7, donor T cells were gated for expression of H-2K^b^, CD45.2, and CD45.1, and analyzed for intracellular expression of IFN-γ and TNF–α following *ex vivo* stimulation with PMA/ionomycin. Data from several experiments were combined and statistical analysis was performed using two-way ANOVA and Student’s *t* test, with p values presented.

Supplementary Figure 4 Quantitative analysis of JAK/STAT and IRF expression and phosphorylation. Quantitative analysis from western blots using Image Lab to normalize to β–Actin, data from 3 independent experiments. **(A)** Phospho and total STAT3. **(B)** Phospho and total JAK1. **(C)** Phospho and total JAK2. **(D)** Total IRF-4. For statistical analysis we used two-way ANOVA and student’s *t* test, p values are presented.

Supplementary Figure 5 Quantitative analysis of tissue BLI. For tissue imaging experiments, allo-HSCT was performed with 10 × 10^6^ WT T cell-depleted BM cells and 1 × 10^6^ FACS-sorted **(A)** CD8^+^ T cells or **(B)** CD4^+^ T cells (from B6-luc or *Itk* ^-/-^ *luc* mice) and bioluminescence imaging of tissues was performed as previously described^20^. Briefly, 5 min after injection with luciferin (10 μg/g body weight), selected tissues were prepared and imaged for 1 min. Imaging data were analyzed and quantified with Living Image Software (Xenogen) and Igor Pro (Wave Metrics, Lake Oswego, OR).
