# Supplementary figures and images for: Targeting Interleukin-2-Inducible T-Cell Kinase (ITK) Differentiates GVL and GVHD in Allo-HSCT
Source: Front Immunol. 2020 Nov 26;11:593863. doi: 10.3389/fimmu.2020.593863 (PMC7726260; doi:10.3389/fimmu.2020.593863)

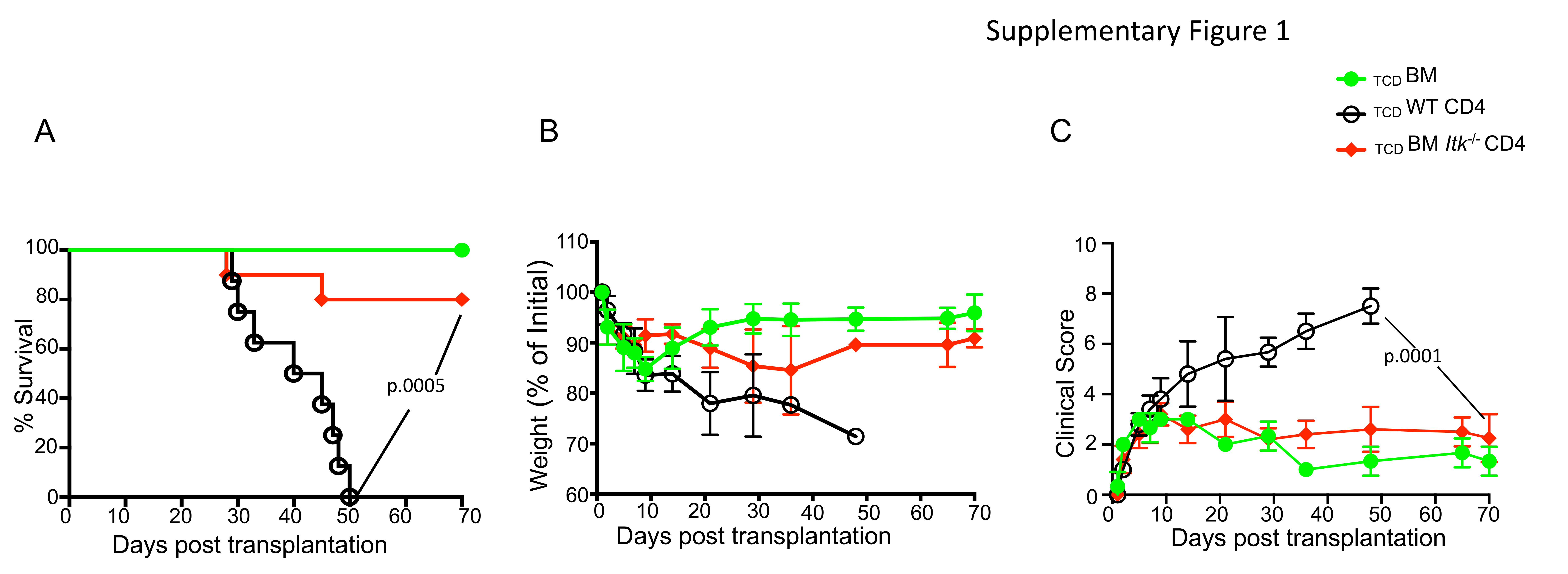

Supplement: Supplementary Figure 1 — Itk-/- CD4+ T cells exhibit attenuated induction of GVHD compared to WT T cells. (A) 10 × 106 TCDBM and 1 × 10^6^ purified WT or Itk-/- CD4+ T cells were transplanted into irradiated BALB/c mice. (A) The mice were monitored for survival, (B) changes in body weight, and (C) clinical score for 70 days post-BMT. For weight changes and clinical score, one representative of 2 independent experiments is shown (n = 3 mice/group for BM alone; n = 5 experimental mice/group for all three groups). The p values are presented. Two-way ANOVA and Student’s t test were used for statistical analysis. [file Image_1.jpg]

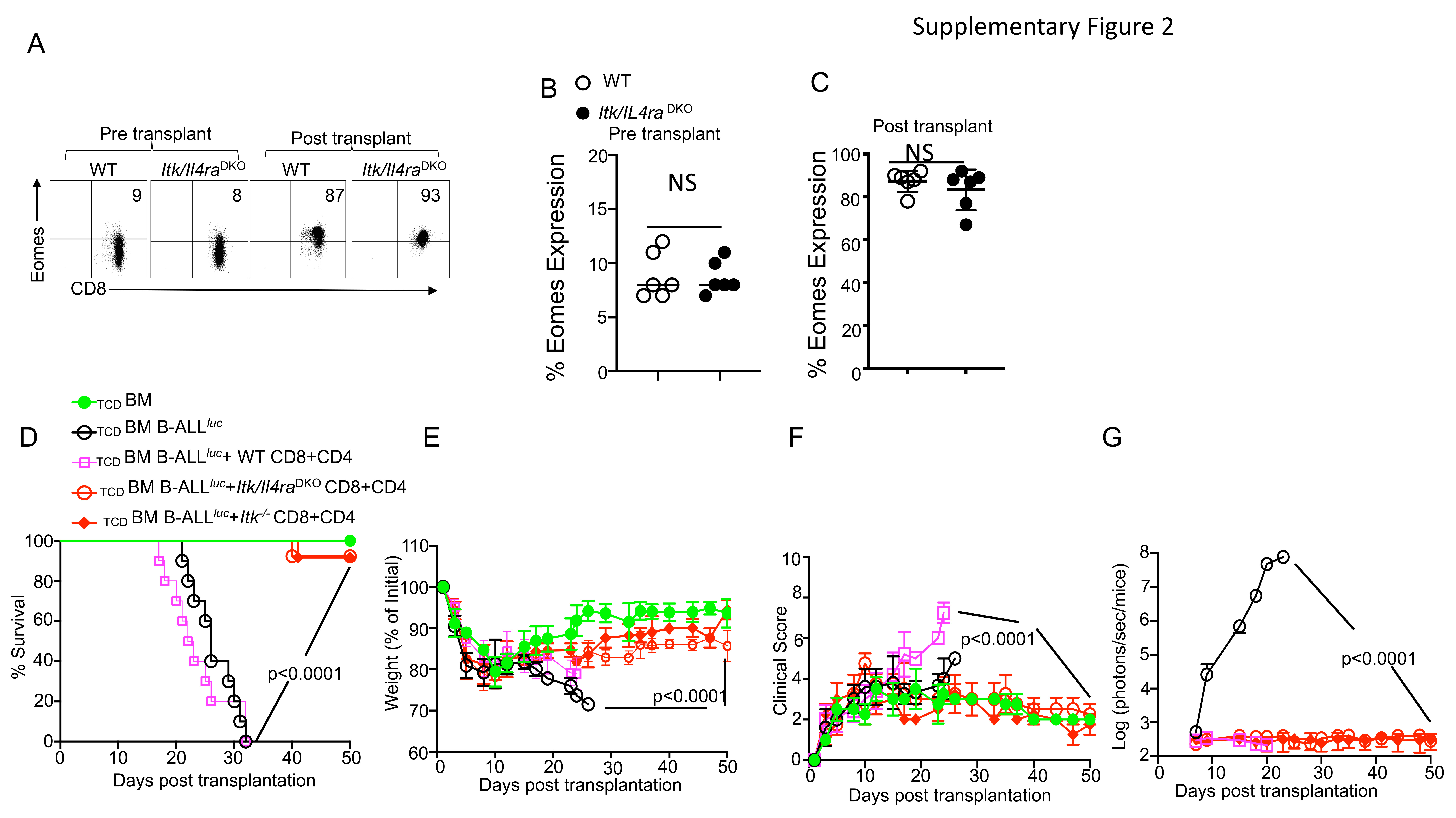

Supplement: Supplementary Figure 2 — IMP T cells are not sufficient for GVHD effect. (A-C) Itk/Il4ra DKO and WT T cells were examined for Eomes expression pre- and post-transplantation. (D) 2 × 10^6^ purified WT and Itk/Il4ra DKO CD8+ T and 1 × 10^6^ purified CD4+ T cells were mixed and transplanted along with 2 × 10^5^ primary B-ALL-luc+ cells into irradiated BALB/c mice. Recipient BALB/c mice were imaged using IVIS 3 times a week. Group one received 10 × 106 TCDBM alone (TCDBM). Group two received 10 × 106 TCDBM along with 2 × 10^5^ B-ALL-luc cells (TCDBM+B-ALLluc). Group three was transplanted 10 × 106 TCDBM with 2 × 10^6^ purified (CD8+ and CD4+) from WT mice and 2 × 10^5^ B-ALL-luc cells (TCDBM+B-ALL luc +WT CD8+CD4). Group four was transplanted 10 × 106 TCDBM and 2 × 10^6^ purified T cells (CD8+ and CD4+) from Itk/Il4ra DKO along with 2 × 10^5^ B-ALL-luc cells (TCDBM+B-ALLluc+ Itk/Il4ra DKO CD8+CD4). Recipient animals were monitored for survival, (E) changes in weight, and (F) clinical score. (G) Leukemia cell growth was monitored as in Figure 1 , and quantitated bioluminescence is shown. One representative of 2 independent experiments is shown (n = 3 mice/group for BM alone; n = 5 experimental mice/group for all three groups. The survival groups were combinations of all experiments. [file Image_2.jpg]

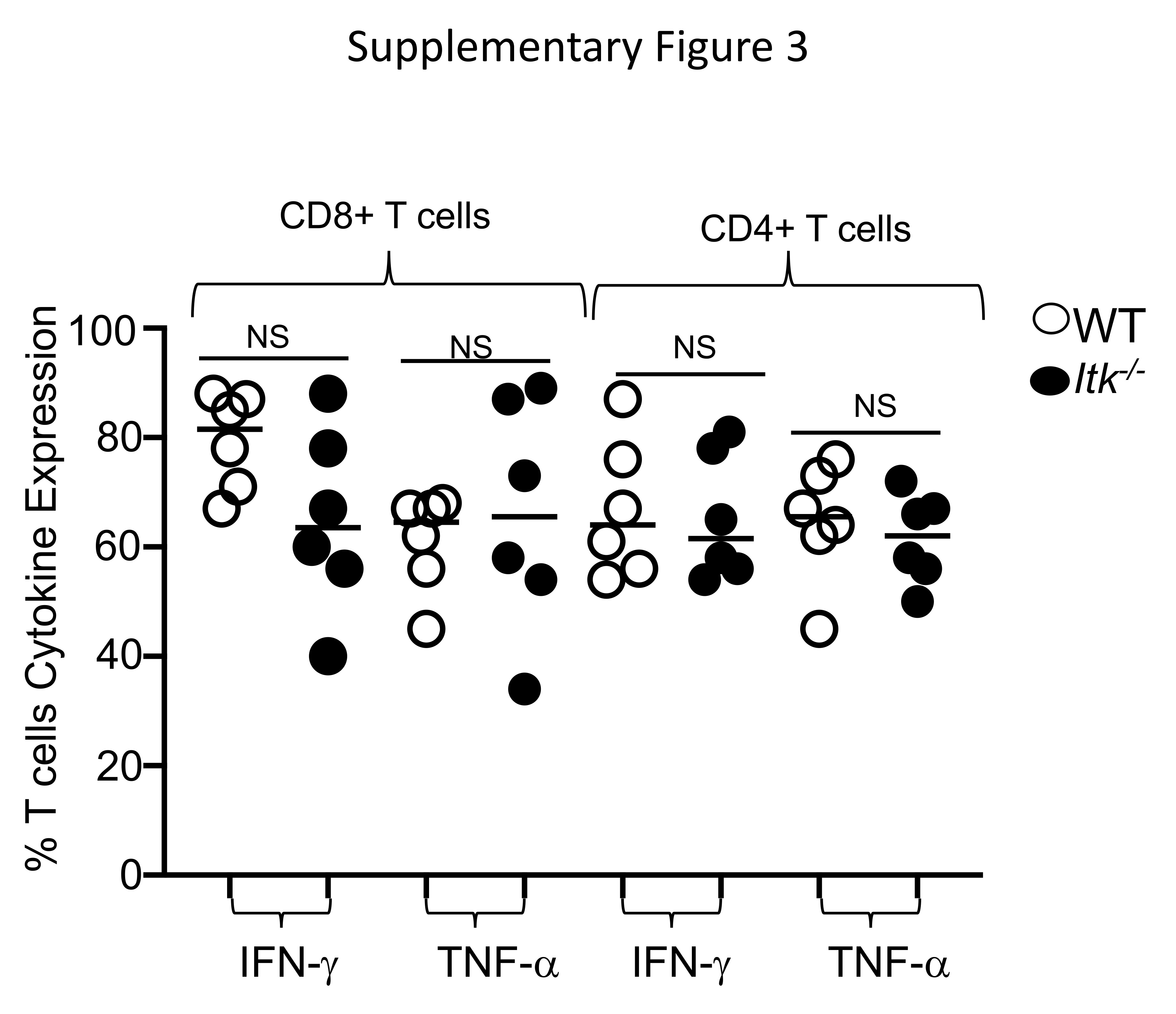

Supplement: Supplementary Figure 3 — Itk-/- T cells are capable of cytokine production. Purified WT and Itk-/- T cells were transplanted into irradiated BALB/c mice. At day 7, donor T cells were gated for expression of H-2Kb, CD45.2, and CD45.1, and analyzed for intracellular expression of IFN-γ and TNF–α following ex vivo stimulation with PMA/ionomycin. Data from several experiments were combined and statistical analysis was performed using two-way ANOVA and Student’s t test, with p values presented. [file Image_3.jpg]

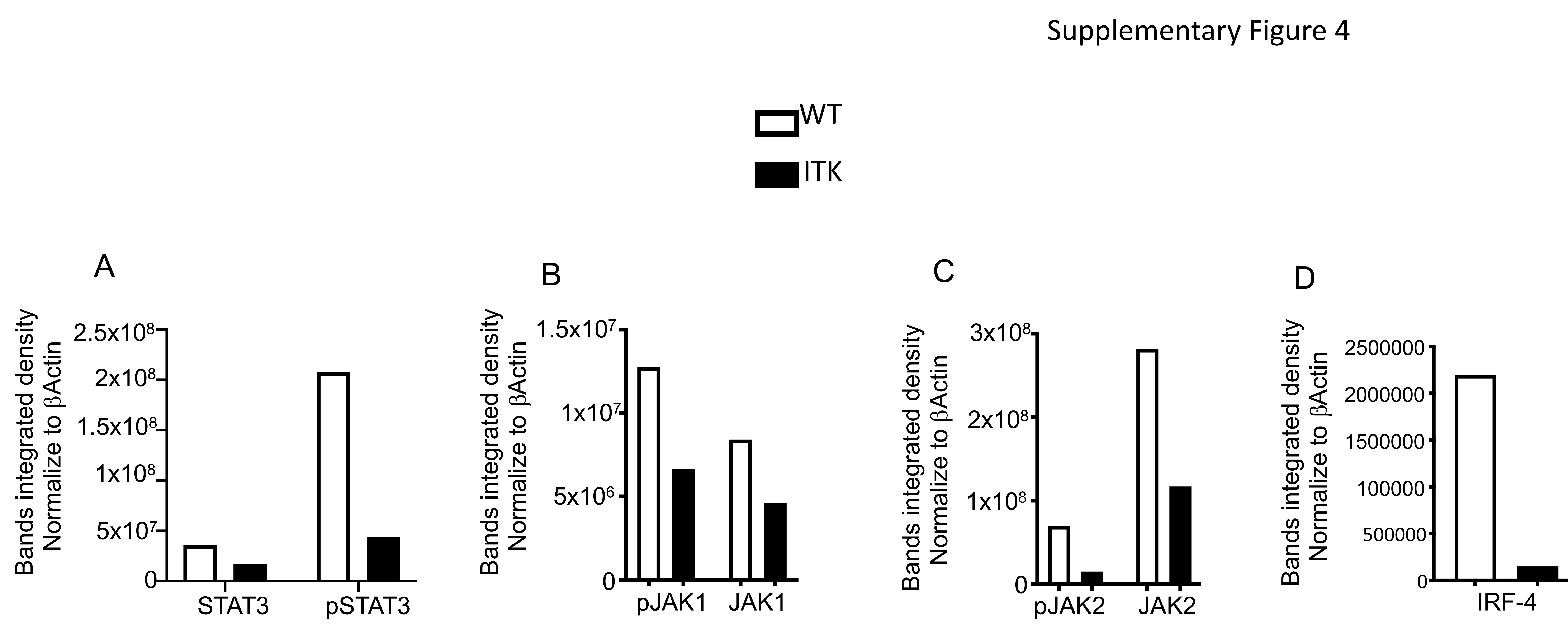

Supplement: Supplementary Figure 4 — Quantitative analysis of JAK/STAT and IRF expression and phosphorylation. Quantitative analysis from western blots using Image Lab to normalize to β–Actin, data from 3 independent experiments. (A) Phospho and total STAT3. (B) Phospho and total JAK1. (C) Phospho and total JAK2. (D) Total IRF-4. For statistical analysis we used two-way ANOVA and student’s t test, p values are presented. [file Image_4.jpg]

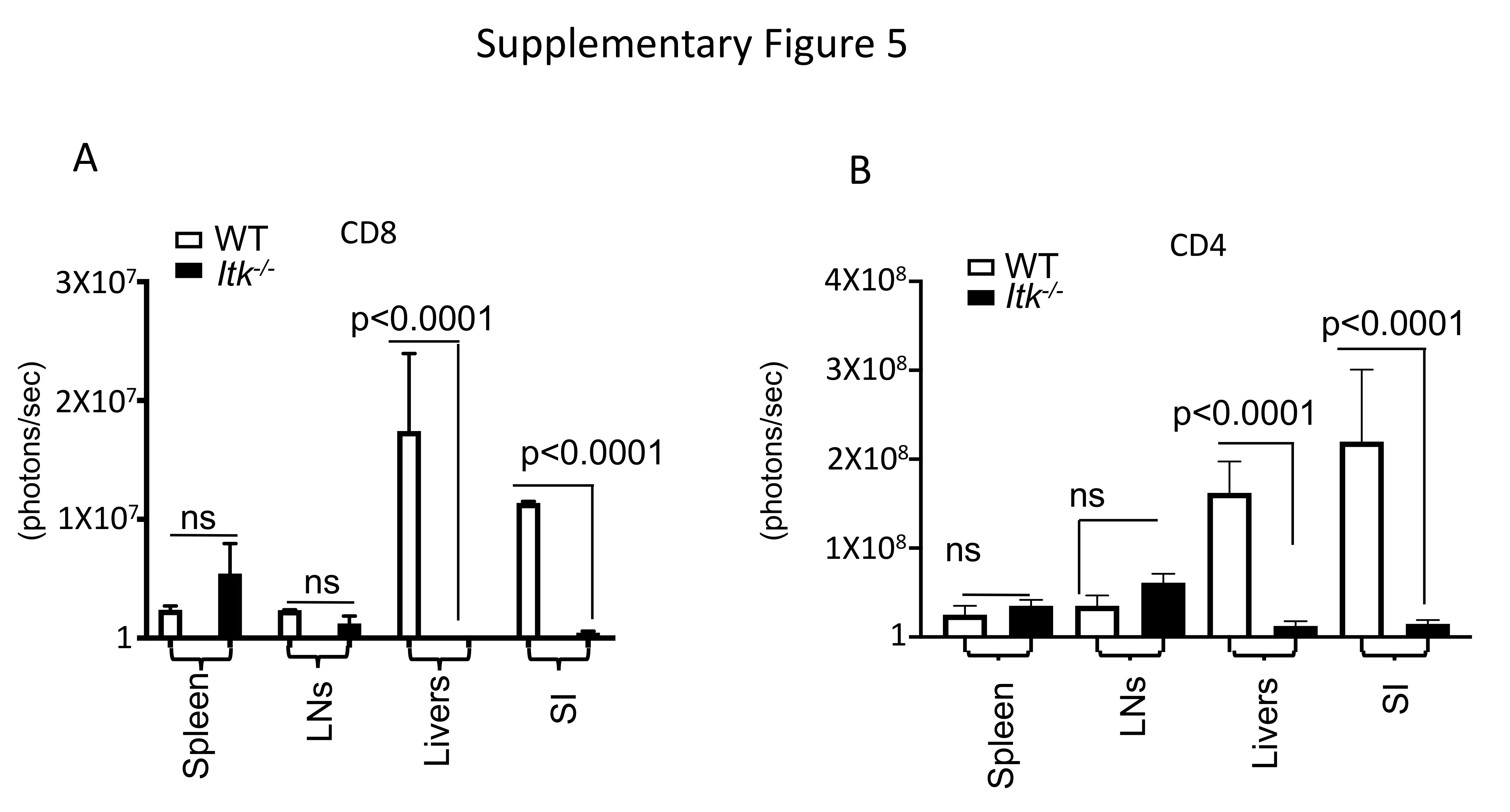

Supplement: Supplementary Figure 5 — Quantitative analysis of tissue BLI. For tissue imaging experiments, allo-HSCT was performed with 10 × 106 WT T cell-depleted BM cells and 1 × 10^6^ FACS-sorted (A) CD8+ T cells or (B) CD4+ T cells (from B6-luc or Itk -/- luc mice) and bioluminescence imaging of tissues was performed as previously described20. Briefly, 5 min after injection with luciferin (10 μg/g body weight), selected tissues were prepared and imaged for 1 min. Imaging data were analyzed and quantified with Living Image Software (Xenogen) and Igor Pro (Wave Metrics, Lake Oswego, OR). [file Image_5.jpg]
